# Bilateral Sequential Spontaneous Anterior Dislocated Intraocular Lens in a Patient With Retinitis Pigmentosa

**DOI:** 10.7759/cureus.26986

**Published:** 2022-07-18

**Authors:** Alex Yee Chau Sim, Yong Meng Hsien, Tang Seng Fai, Mushawiahti Mustapha, Wan Haslina Wan Abdul Halim

**Affiliations:** 1 Ophthalmology, Universiti Kebangsaan Malaysia Medical Centre, Kuala Lumpur, MYS; 2 Ophthalmology, Universiti Kebangsaan Malaysia Medical Centre, Kuala lumpur, MYS

**Keywords:** bilateral eye, cornea decompensation, pupillary block glaucoma, anterior dislocated intraocular lens, retinitis pigmentosa

## Abstract

Retinitis pigmentosa is one of the risk factors for intraocular lens dislocation post cataract surgery which can lead to many complications. A 64-year-old Chinese female with bilateral pseudophakia and retinitis pigmentosa was referred for the continuation of care in 2009 with baseline visual acuity of hand movement bilaterally due to the retinitis pigmentosa. The cataract surgeries with posterior chamber intraocular lens (PCIOL) implantation in her early 50s were uneventful.

In 2011, her right eye PCIOL dislocated anteriorly into the anterior chamber spontaneously and touched the cornea. It was complicated with bullous keratopathy and corneal decompensation. Intraocular pressure (IOP) was normal. PCIOL explantation, anterior vitrectomy and surgical peripheral iridotomy were performed. However, the cornea remained decompensated postoperatively.

Her left eye was stable until 2019 when she developed acute angle closure secondary to complete anterior dislocation of PCIOL with pupillary block glaucoma. She underwent left eye PCIOL explantation, anterior vitrectomy and surgical peripheral iridotomy when IOP was optimised medically. Finally, both eyes were left aphakic due to poor prognosis with light perception (PL) vision, IOP was stable on single topical antiglaucoma and bilateral decompensated corneas were maintained with topical hypertonic saline. This case highlights the different serious sequelae of bilateral eyes in an unfortunate retinitis pigmentosa patient.

## Introduction

Retinitis pigmentosa is a group of hereditary disorders affecting the photoreceptors and retinal pigment epithelium [1]. Patient with retinitis pigmentosa frequently has night blindness, visual field loss, central vision dysfunction, rod-cone dystrophy and abnormal electroretinogram (ERG) [2]. The fundus is typically characterised by waxy pale optic disc, retinal arteriolar attenuation and bony spicule pigmentation, especially at the peripheral retina. Additionally, cataract is commonly observed among them at a relatively young age, especially posterior subcapsular cataract [1,3]. Other ocular findings include keratoconus and glaucoma.

Interestingly, retinitis pigmentosa is one of the risk factors for intraocular lens (IOL) dislocation post cataract surgery which can lead to many complications [1,3,4]. Hence, we would like to report a case of bilateral sequential spontaneous anterior dislocated posterior chamber intraocular lens (PCIOL) in a patient with retinitis pigmentosa.

This article was previously presented as a meeting poster at the 33rd Asia-Pacific Association of Cataract and Refractive Surgeons (APACRS)-Singapore National Eye Centre (SNEC) 30th Anniversary Virtual Meeting on July 30-31, 2021.

## Case presentation

A 64-year-old Chinese female with bilateral pseudophakia and retinitis pigmentosa was referred to a private centre for the continuation of care in 2009 with baseline best corrected visual acuity of hand movement (HM) bilaterally due to the retinitis pigmentosa. The cataract surgeries with PCIOL implantation in her early 50s were uneventful bilaterally. Further questioning, she denied any history of wearing spectacles prior to that.

**Figure 1:**
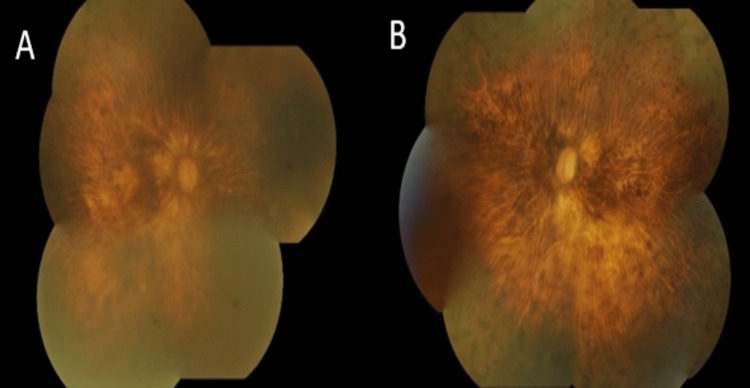
The fundi of this patient showed extensive bony spicule which involved macula with pale optic disc in both the (A) right eye and (B) left eye.

Both fundi showed extensive bony spicules with pale waxy optic disc and attenuated arterioles (Figure 1). Intraocular pressure (IOP) was normal in both eyes. She was started on eyedrop brimonidine-P 0.15% to preserve the remaining optic nerve bilaterally.

**Figure 2:**
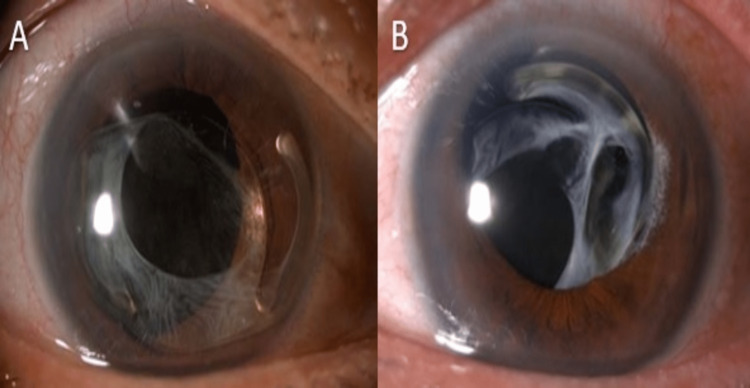
(A) Anterior dislocation of posterior chamber intraocular lens (PCIOL) over her right eye in 2011. (B) Mild inferior subluxated PCIOL over her left eye. However, it was still within the visual axis.

Two years later in 2011, the patient presented with a right eye spontaneously dislocation of PCIOL together with the capsular bag into the anterior chamber (Figure 2A). She denied pain or red eye. There was no history of ocular trauma. It was a single-piece PCIOL. The PCIOL was not broken but it touched the cornea and was complicated with bullous keratopathy and corneal decompensation. The endothelial cell count was too low to maintain the corneal clarity. The IOP was within the normal range. We proceeded with right eye lens explantation, anterior vitrectomy, and surgical peripheral iridotomy. Meanwhile, her left eye showed mild subluxated PCIOL inferiorly (Figure 2B). However, it did not compromise the cornea. She was not keen on intervention in the left eye at that time.

Post-operatively, her right eye visual acuity was a light perception (PL) in all quadrants. We decided to leave the right eye aphakia due to a guarded prognosis. The cornea remained decompensated. On the other hand, her left eye remained stable with vision HM.

**Figure 3:**
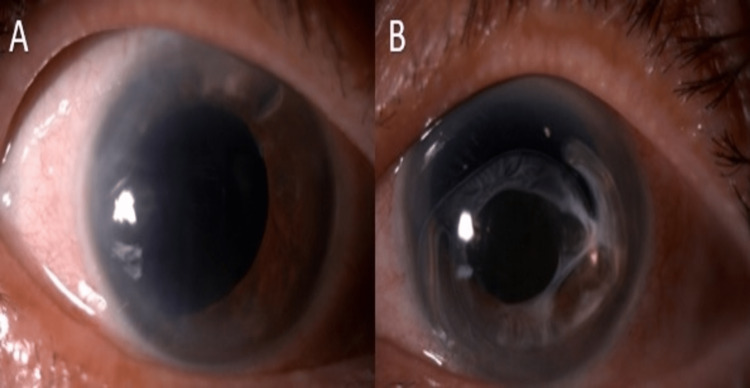
(A) Aphakia with the decompensated cornea in the right eye. (B) Anterior dislocation of posterior chamber intraocular lens (PCIOL) complicated with IOL-pupillary block glaucoma in the left eye.

Ten years after initial cataract surgery in 2019, she developed sudden left eye pain and redness. She was diagnosed with left eye acute angle closure with IOP of 50mmHg and oedematous cornea, secondary to complete anterior dislocation of PCIOL that was complicated with IOL-pupillary block glaucoma (Figure 3B). She denied any history of trauma as well. Gonioscopy was attempted but poor view. Meanwhile, her right eye remained aphakic and decompensated cornea (Figure 3A).

IOP lowering agents were given extensively to the left eye, including topical latanoprost 0.005% ON, timolol 0.5% BD and brimonidine-P 0.15% BD along with oral acetazolamide 250mg QID and Slow K 600mg BD. Topical dexamethasone 0.1% QID was given to control the inflammation. After the IOP was stabilized medically, she underwent left eye PCIOL explantation, anterior vitrectomy and surgical peripheral iridotomy uneventfully. Similarly, this eye was left aphakic due to a guarded prognosis.

**Figure 4:**
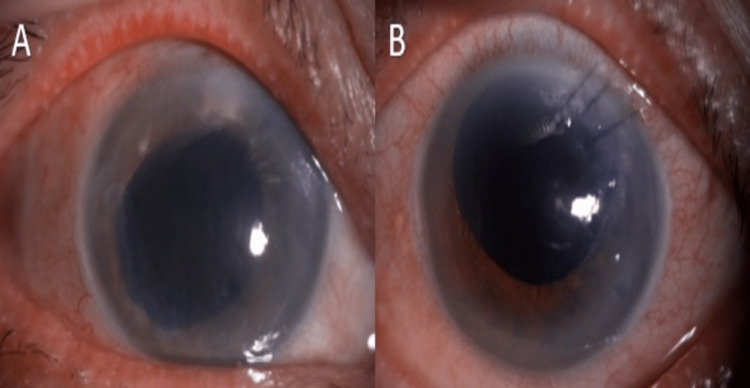
Both (A) right eye and (B) left eye were aphakia with the decompensated cornea.

**Figure 5:**
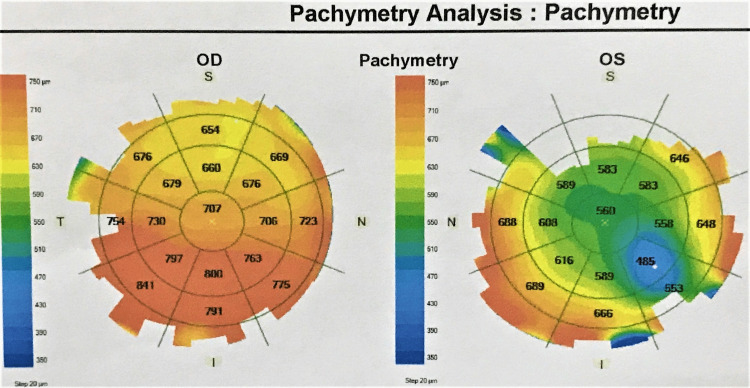
Both eyes showed corneal oedema and decompensation, right eye (OD) was more severe than the left eye (OS). OD: oculus dextrous; OS: oculus sinister; μm: micrometer

Recent follow-up in October 2021 reviewed bilateral vision PL. Both eyes were aphakia with the decompensated cornea (Figures 4A, 4B). IOP was stable with single topical antiglaucoma (eyedrop brimonidine-P 0.15% BD) in both eyes. Topical hypertonic saline was given for the persistent decompensated corneas as shown by pachymetry (Figure 5).

## Discussion

There is some common complications post cataract removal being observed among retinitis pigmentosa patients. This includes anterior capsular contracture, posterior capsular opacification, and cystoid macula edema [1,5]. On the other hand, the prevalence of PCIOL dislocation post cataract surgery among them was only about 9%-10% [3,5].

Multiple mechanisms have been postulated for PCIOL dislocation post cataract surgery among retinitis pigmentosa patients. This includes trauma, capsule contraction, zonular dehiscence or weakness and Nd:YAG laser capsulotomy [6].

Hayatsi et al. also reported a significant reduction in the mean area of the anterior capsular opening post cataract operation in retinitis pigmentosa patients. He proposed that the blood-derived cytokines maybe are more abundant in the aqueous humour of these groups of patients due to the disrupted blood ocular barrier. These cytokines will then stimulate the lens epithelial cell proliferation that led to capsule fibrosis eventually [7].

In terms of zonular weakness, Samuel et al. showed that 10% of the eyes had phacodonesis discovered during cataract removal and 5% detected only after cataract removal. The postulation for zonular weakness is secondary to the direct damage from the toxic substances of degenerated retina among retinitis pigmentosa patients. He also suggested that the mechanism for zonular weakness among them can be due to the anterior capsular contraction [8]. The proliferation of lens epithelial cells may increase IOL-capsular bag mass which will contribute to the increased zonular stress subsequently [9].

Smith et al. reported a case of PCIOL dislocation following Nd:YAG laser capsulotomy where he believed it is a combination of severe anterior capsule fibrosis, zonule weakness, and extensive vitreous degeneration which are strongly associated with retinitis pigmentosa patients [10].

Meticulous steps should be planned prior to cataract surgery in retinitis pigmentosa patients. A larger size of capsulorhexis is suggested [1,7]. Capsular tension ring (CTR) has been proposed intra-operatively albeit dislocated IOL postoperatively is still being reported in some cases [6]. Furthermore, some ideal techniques can be used intraoperatively to reduce the stress of zonules, for example, chopping technique with gentle hydrodissection may be preferred [4,8,9]. Alternative IOL placement such as anterior chamber intraocular lens (ACIOL) and scleral fixated IOL can also be considered instead of in-the-bag [6]. Silicone IOL should be avoided as it will induce capsulorhexis contracture the most [9].

The indications for surgical intervention in subluxated or dislocated IOL include glaucoma, risk of corneal decompensation, diplopia and retinal detachment [9]. The decision for secondary lens implantation would depend on the patient’s factors and the prognosis of the eyes.

## Conclusions

Retinitis pigmentosa patients are susceptible to delayed subluxation or dislocation of IOL even with uneventful cataract surgeries, due to its underlying zonulopathy. Meticulous operative steps should be always planned carefully prior to cataract operation. Intraoperatively, care should be taken to reduce the risk of zonulopathy as undesired sequelae following dislocated PCIOL will affect other ocular structures on top of the impairments by unfortunate retinitis pigmentosa. These mentioned risks should be informed to the patients.
